# DNA/RNA Electrochemical Biosensing Devices a Future Replacement of PCR Methods for a Fast Epidemic Containment

**DOI:** 10.3390/s20164648

**Published:** 2020-08-18

**Authors:** Manikandan Santhanam, Itay Algov, Lital Alfonta

**Affiliations:** Departments of Life Sciences, Chemistry and Ilse Katz Institute for Nanoscale Science and Technology, PO Box 653, Ben-Gurion University of the Negev, Beer-Sheva 8410501, Israel; manikandan.mb@gmail.com (M.S.); algov@post.bgu.ac.il (I.A.)

**Keywords:** electrochemical DNA sensor, nucleic acid sensor, signal amplification, DNA, RNA, pathogen sensing

## Abstract

Pandemics require a fast and immediate response to contain potential infectious carriers. In the recent 2020 Covid-19 worldwide pandemic, authorities all around the world have failed to identify potential carriers and contain it on time. Hence, a rapid and very sensitive testing method is required. Current diagnostic tools, reverse transcription PCR (RT-PCR) and real-time PCR (qPCR), have its pitfalls for quick pandemic containment such as the requirement for specialized professionals and instrumentation. Versatile electrochemical DNA/RNA sensors are a promising technological alternative for PCR based diagnosis. In an electrochemical DNA sensor, a nucleic acid hybridization event is converted into a quantifiable electrochemical signal. A critical challenge of electrochemical DNA sensors is sensitive detection of a low copy number of DNA/RNA in samples such as is the case for early onset of a disease. Signal amplification approaches are an important tool to overcome this sensitivity issue. In this review, the authors discuss the most recent signal amplification strategies employed in the electrochemical DNA/RNA diagnosis of pathogens.

## 1. Introduction

Rapid, specific and sensitive detection is a goal in emerging biosensor technology. Detection of pathogens using their genomes becomes a central strategy due to advancement of nucleic acid sequencing technologies [1]. Nucleic acid-based detection of pathogens provides more flexibility compared to other biomolecules, as they are present in all living organisms, while every organism or a virus encode their genes with a distinct genome and sequences. However, access to the DNA/RNA sequences in a viral pathogen is not straight forward; they are buried inside bilayers of lipids and proteins of the virion particle. Thus, DNA/RNA should be efficiently extracted from a potential sample. Additionally, the copy number of a given virion may vary, depending on the stages of the infection, virulence and the host cells’ replication efficiency. In the case of RNA targets, RNA is reverse transcribed to complementary DNA using reverse transcriptase and then it is quantified.

Currently, quantitative real time-polymerase chain reaction (qPCR) is a standard method where a fluorescent signal is coupled with DNA polymerase chain reaction for quantification of DNA [2,3,4,5,6]. Though this method affords the detection of the presence of 1–10 copies/mL of DNA sample, PCR is still restricted to professional laboratories due to the need for specialized instrumentation [7,8]. To simplify DNA detection for point-of-care testing, other alternative approaches are being developed, namely, colorimetric [9], microfluidic platform based optical detection [10] and electrochemical methods [11]. Among these methods, electrochemical methods are ultrasensitive and well established [12]. In electrochemical DNA sensors, nucleic acid hybridization is coupled with the electrochemical reaction for selective detection of target DNA [13,14]. However, electrochemical methods may not be employed directly for the detection of a single copy of DNA sensing in biological samples. Thus, signal amplification approaches are employed to increase the sensitivity and selectivity towards the sensing of low concentration targets [15]. Additionally, in terms of the possibility to miniaturize as well as to make a quantitative measurement, electrochemical sensors is currently the most promising route for laboratory as well as for the point-of-care approaches [16,17].

Comprehensive reviews on bacterial, protozoan, viral and clinical diagnostics through a variety of electrochemical systems have been published before [18,19,20]. Additionally, different aspects of DNA electrochemical sensors, such as novel materials and electroanalytical methods, were reviewed in detail [21,22,23,24,25,26]. In this review, developments in signal amplification approaches for enhanced detection of DNA–DNA or DNA–RNA hybridization events using electrochemical approaches are discussed and directed to non-specialized readers. Specifically, signal amplification approaches with synthetic and/or real samples of pathogens are discussed.

## 2. Signal Transduction

**Figure 1:**
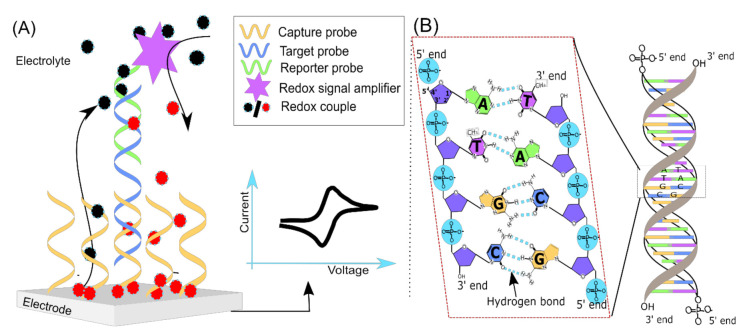
Nucleic acids electrochemical biosensor general principles. (**A**) A sandwich type genosensor model: A capture probe is employed to capture the target (DNA/RNA) from the solution phase to the electrode surface. The electrode bound target DNA is quantified indirectly by binding the reporter probes conjugated with a redox signal amplifier. The redox signal amplifier could be an enzyme or a nanomaterial, which produces the redox-active molecules. The redox-active molecules undergo an oxidation/reduction reaction, which is then quantified as an electrical response (current–voltage response) using electrochemical analytical methods. The whole strategy depends solely on hybridization efficiency between the nucleic acid probes and the target molecules (RNA/DNA/PNA). In this approach, target DNA does not need any modification. (**B**) The double-helical structure of DNA and Watson and Crick base pairing in DNA. DNA consists of two strands. The two strands are held together by complementary base pairing between the bases, i.e., hydrogen bonds (A with T and G with C). Two hydrogen bonds attach A to T; three hydrogen bonds attach G to C. High temperature can denature the double-stranded DNA into single-strands. These complementary single-stranded DNAs can specifically rehybridized to form a double-stranded helix by reducing the reaction temperature.

DNA electrochemical biosensors consist of (i) a DNA recognition element where the target DNA is recognized by a probe/capture DNA(s) strand and (ii) a signal transduction part where the molecular recognition event is translated into an electrical response by an electrochemical reaction [27] (Figure 1A). Two complementary strands of DNA specifically hybridize with each other; thus detection of this hybridization event has become a central theme in biosensor studies [14,26]. The DNA hybridization is based on Watson and Crick base pairing rules (Figure 1B), i.e., specific hydrogen bond formation between two (target DNA and probe DNA) complementary single strands of DNA (ssDNA) [28]. In a sensor detection scheme, ssDNA(s) specifically hybridizes with a target DNA sequence that is being employed as a probe(s): a capture probe used to attach the target DNA to the surface of materials and/or a reporter probe labeled with signaling molecules, e.g., redox-active molecules. The hybridization reaction occurs in solution (homogeneous) or at an electrode/transducer surface (heterogeneous) [12]. The advantage of hybridization in the solution phase is that it is well controlled using known properties such as melting temperature (T_m_) and ionic strength of the buffer [14,29]. In the case of hybridization on an electrode surface, the probe DNA is attached to the electrode surface in such a way that the sequence is available to target DNA in the hybridization solution. Additionally, non-specific interactions of DNA with the electrode should be avoided along with other optimization processes such as surface coverage and incubation time [30,31], these will not be discussed herein.

The DNA hybridization product could be selectively and electrochemically quantified on the electrode surface. DNA adsorbed on electrode surfaces are stable below guanine oxidation potential (Table 1), this stability is advantageous for immobilization of DNA directly on the electrode surface. To make a quantitative measurement, the DNA hybridization event is coupled with electrochemical reactions, in a way that a probe-target complex increases/decreases a coupled redox reaction at the electrode surface. It can also be achieved by measuring the changes in the electrode/electrolyte interface properties due to a DNA hybridization event. In general, DNA sensors are categorized into several types based on what kind of probe DNA is being used (label-free/labeled) and how is signal transduction achieved (reagent-free or reagent-dependent). The detailed information on DNA sensor history, principles and fabrication approaches is thoroughly reviewed elsewhere [12,16,26,32,33]. In this review, approaches specific to signal amplification that involves pathogenic DNA/RNA detection is reviewed.

## 3. Signal Amplification Approaches for the DNA/RNA Electrochemical Sensor

DNA hybridization is a selective process where even a single mismatch between the target and probe can be differentiated in most cases [14,29]. However, often, sensitive detection of the capture-target hybridization event is challenging. Since clinical samples may have very low copies of a pathogen in the early stages of infection, as low as 1–10 colony forming units (CFU/mL) [8]. Electrochemical methods are inherently very sensitive to detect even fM target DNA concentrations [41,42,43,44,45,46,47]. To overcome the limitation of instrumentation requirement for sensor deployment, signal amplification strategies have been investigated and developed to enhance the electrochemical signal (Table 2). Amplified signals are quantified using electrochemical analytical techniques such as chronoamperometry (CA), differential pulse voltammetry (DPV), square wave voltammetry (SWV), cyclic voltammetry (CV) and electrochemical impedance spectroscopy (EIS; Table 2). Signal amplification methods in combination with electrochemical analytical techniques were demonstrated for the detection of femto- and attomolar concentrations of DNA. Herein, we illustrate and discuss several signal amplification methods that were reported for pathogen detection. The methods are categorized into (i) enzyme mediated signal amplification, (ii) nanomaterials-based approaches and (iii) nucleic acid-based approaches.

### 3.1. Enzyme Mediated Signal Amplification

**Figure 2:**
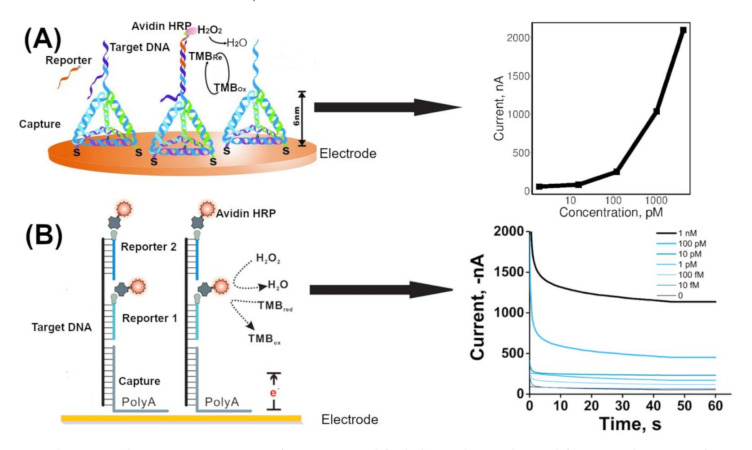
Schematic presentation of an HRP amplified electrochemical signal for DNA detection yth of enzyme molecules for the electrochemical signal. (**A**) DNA tetrahedral nanostructure for enhanced signal detection on gold surfaces [71]. (**B**) PolyA–gold surface interaction for immobilization of capture DNA, which was combined with multiple reporter probes and was attached to multiple HRP enzyme copies for signal amplification [50]. Adapted with permission from cited sources.

The use of an enzyme for signal amplification can aid in increasing sensitivity of a sensor in that a single recognition event that can be sensed only stoichiometrically could be transduced and recycled several times by the biocatalytic reaction mediated by an enzyme that is coupled to this recognition event. Several enzymes have been strategically conjugated with DNA hybridization complexes to amplify electrochemical signals. When an enzyme is tagged with a probe DNA, each hybridization event is coupled to an enzyme molecule. Each enzyme can produce multiple (10–1000) fold higher redox-active products. This can result in a multifold enhanced redox current at the electrode surface. Horseradish peroxidase (HRP) [70], alkaline phosphatase [64], lipase [54], invertase [11] and glucose oxidase [68] were successfully employed for signal amplification of pathogen detection studies. Different methods have been employed in signal amplification approaches to detect a low copy number of target DNA on the electrode surface. Application of magnetic beads and advancement in functionalization of nano/micro bead structures provides the ability to specifically enrich the target DNA from the background matrix components. Bioconjugates (e.g., biotin-avidin) are utilized as molecular binders with high affinity for building a network of molecular conjugations. Alzate et al. demonstrated a magnetic bead-based approach to quantify the Zika virus [51]. First, a biotinylated capture probe was immobilized on streptavidin-coated magnetic beads. Second, the target was prehybridized with the Digoxigenin (Dig)-labeled reporter probe and then added to the capture probe-coated magnetic beads to hybridize. Third, the reporter probe was recognized by an anti-Dig monoclonal antibody labeled with horseradish peroxidase (HRP). The final bead complex was magnetically attracted to the surface of a screen-printed electrode. H_2_O_2_ and 3,3′,5,5′-tetramethylbenzidine (TMB) were added as the HRP substrate. This strategy achieved the detection of 10 pM synthetic target ssDNA. Dong et al. reported the use of a DNA tetrahedral nanostructure-based electrochemical biosensor to detect avian influenza A (H7N9) virus [49]. The tetrahedral nanostructure was used as a biomolecule-confined surface to increase molecular recognition at the biosensing interface (Figure 2A) [71]. First, the DNA tetrahedral structure was immobilized onto a gold electrode surface via an Au-thiol bond. A single strand part of the tetrahedral DNA acted as the capture DNA to hybridize with a target ssDNA. The capture-target sequence was hybridized with a biotinylated reporter DNA sequence. Then streptavidin-horseradish peroxidase was introduced to bind the biotinylated reporter–target DNA hybrids. The reduction current for HRP oxidized TMB substrate was measured using an amperometric method. When this sensor was used for the detection of PCR products (ssDNA) amplified from cDNA isolated from positive patients, the 1.2–1200 pM range was detected. It was also shown that 1–5 cycles of asymmetric PCR generated enough target DNA for the experiment. Wang et al. reported a multiple-reporter probe approach for detection of the 16S rRNA gene of different bacteria [50] (Figure 2B). In this approach, A high-adsorption affinity of the polyA tail towards the Au surface was used to immobilize the molecular recognition complex on an Au electrode [72]. First, the target DNA was hybridized with a multiple biotinylated reporter probe. Second, the prehybridized target-reporter probe was hybridized with the capture probe immobilized at the Au electrode. The capture DNA sequence was designed to have a polyA tail. Then, an Avidin-HRP conjugate was bound to the biotinylated groups of reporter probes in the hybrid complex. The usage of multiple-reporter probes enhanced the number of HRP molecules per hybridization event [73]. The detection range was reported to be 10 fM to 1 nM of synthetic targets. The sensor was also successfully tested for specific detection of denatured genomic DNA from bacterial samples.

Walter et al. presented a simple approach in which signal amplification was achieved by redox cycling of *p*-aminophenol phosphate (*p*-AP) using nicotinamide adenine dinucleotide [53]. A molecular recognition complex based on sandwich-type hybridization and reporter probe was tagged with alkaline phosphatase. The electrochemically inert *p*-AP was converted to an electrochemically active form of *p*-AP by tagged alkaline phosphatase. Enzymatically generated *p*-AP was electro-oxidized at an Au electrode to p-quinone imine (*p*-QI) and in the presence of NADH, *p*-QI was reduced back to *p*-AP, which was reoxidized on the electrode. This approach overcame the drawbacks associated with the stability of *p*-AP. It has allowed reaching a detection limit of 1 pM of target DNA. When it was applied for the monitoring of the 16S rRNA of *E. coli* pathogenic bacteria it had a detection limit of 250 CFU μL^−1^.

**Figure 3:**
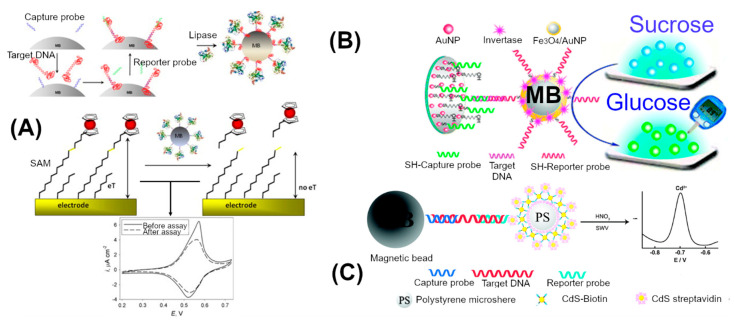
Schematic presentation of an electrochemical signal amplification for DNA detection. (**A**) DNA sandwich with a lipase labeled reporter probe for detection of *Lactobacillus brevis* DNA. Lipase was designed to bind with capture and target molecular recognition elements. During electrochemical analysis, lipase cleaves off the ferrocene from 9-mercaptononyl, 4-ferrocene aminobutanoate monolayer over the electrode surface. This results in the reduction of the observed current using cyclic voltammetry [74]. (**B**) Multiple invertase copies coated magnetic bead was conjugated with each capture and target molecular recognition element. The invertase was used to convert sucrose to glucose. Glucose was detected by a glucose meter. This system was reported for detection of HIV DNA [11]. (**C**) Similar to invertase, CdS coated polystyrene bead was used as a signal amplifier for the detection of urinary tract pathogens [61]. The Cds nanoparticle bound to the molecular recognition element was dissolved in the acid solution and resulting cadmium ions were quantified electrochemically. Adapted with permission from cited sources.

The signal turn-off mode system was employed for enhanced detection with enzyme-mediated signal amplification. In a signal-off mode, the current signal decreases as a function of DNA concentration. Shipovskov et al. demonstrated lipase chemistry to detect the low amount of target DNA in a signal turn-off mode (Figure 3A) [54]. They established an ester bond containing self-assembled monolayer (SAM) of 9-mercaptononyl, 4-ferrocene aminobutanoate (Fc-alkanethiol ester) on a gold surface, which exhibited high surface redox current using CVs [74]. Lipase was used to cleave the ester bond to remove ferrocene (Fc), a redox-active molecule, from a SAM layer, which resulted in a decrease in current in this system. First, a streptavidin-coated magnetic bead was decorated with biotinylated capture DNA. Then, step by step, it was allowed to react with a target DNA, biotinylated reporter probe and a streptavidin-lipase conjugate. At the end of the hybridization step, the final complex was immobilized on a magnetic bead, which was then applied on an Fc-alkanethiol ester SAM on a gold electrode. The lipase coupled to the DNA recognition complex removed the Fc from the SAM. This resulted in a decrease in the CV peak current. The lowest detected signal peak was 4 fM of the synthetic target DNA. Further, the assay could be used for the detection of down to 16 aM of denatured RNA and their cDNA copies prepared from *Lactobacillus brevis*. In a similar turn off mode, Luo et al. has employed exonuclease III for the detection of low levels of *E. coli* in milk samples [64]. First, a capture DNA was immobilized through its 5′-end. Second, the target DNA was hybridized with the capture probe to form a double-stranded structure, which resulted in a blunt end at the 3′-end of the capture probe. Then Exo III, an exonuclease, was introduced to catalytically remove the mononucleotides from the 3′-hydroxyl termini of DNA duplexes. The Exo III activity degraded the capture DNA strand and released the target DNA. The released target was recycled for more capture DNA degradation. After a fixed duration of treatment with the Exo III treatment, the capture DNAs that were not degraded on the sensor surface hybridized with the biotinylated reporter probe. The reporter probe was linked to streptavidin-alkaline phosphatase to produce an enzymatic electrochemical guanine signal for quantitative detection of Enterobacteriaceae bacteria. Using this approach about 40 CFU/mL of *E. coli* was electrochemically detected where a single strand PCR product was used as a target.

Screen-printed electrodes, which require a small sample volume, are widely employed in sensor development studies. However, the electrochemical response analysis for the screen-printed electrode is still limited to high-end laboratory-based instrumentation. Instead of conventional laboratory-based electrochemical techniques, the commercial glucose meter was also successfully demonstrated for the detection of pathogen’s DNA. Xu et al. demonstrated multiple invertase-mediated signal amplification and the use of a glucometer as an electrochemical device for the detection of HIV DNA [11] (Figure 3B). First, a mixed layer of thiolated capture probes and 6-mercaptoethanol were self-assembled on the AuNPs via thiol–Au attachment. The capture probe coated AuNPs were applied on the glassy carbon electrode. Then target DNA was hybridized with capture probes. The reporter probe was tagged with multiple-invertase coated-Fe_3_O_4_/AuNPs using thiol chemistry. Hybridization of invertase coated reporter probes has led to massive quantities of invertase on the electrode surface. Glassy carbon electrode was used to characterize the loading of the probe and target DNA using [Fe(CN)_6_^3−/4−^] redox couple. To quantify the target DNA, sucrose was introduced onto the glassy carbon electrode surface containing a molecular recognition complex. Upon introducing sucrose, invertase converted the sucrose into glucose molecules, which were measured by a glucometer. Due to several numbers of tagged invertase per hybridization event and its high turnover number, glucose in millimolar concentrations was produced. Using this approach, about 0.5 pM to 1 nM concentration of synthetic HIV DNA was detected using a standard glucose meter sensitivity range.

### 3.2. Nanomaterial Enhanced Signal

The nanomaterials are used as reporter molecules and high surface area materials for high loading of probe DNA. In case of a nanomaterial as a reporter, metal-based nanoparticles were tagged with DNA for hybridization. Xiang et al. reported CdS quantum dot decorated polystyrene (PS-(CdS)_4_) as a signal amplifier for the detection of urinary tract pathogen (Figure 3C) [61]. PS-(CdS)_4_ was built using biotin and streptavidin functionalized PS and CdS nanoparticles. First, the biotin-capture probe was immobilized on streptavidin-magnetic beads and then incubated with the target DNA. The magnetic bead–target complex was then hybridized with a reporter probe, which was immobilized on polystyrene-CdS spheres (PS-(CdS)_4_). The resulting complex was selectively separated by magnetic separation and was treated with nitric acid to dissolve the CdS nanoparticles. Cd ions in the solution were measured using square wave voltammetry. Using this approach, 0.5 fM to 10 pM of synthetic DNA was detected. In a similar metal nanoparticle-based approach, Zhang et al. demonstrated detection of multiple pathogens using nanoparticle-based biobarcoded electrochemical sensors [75]. Each pathogen-specific probe sequence was tagged to specific nanoparticles. The detection limit of bio-barcoded DNA sensor was 0.5 ng/mL for the insertion element (*Iel*) gene of *Salmonella enteritidis* using CdS, and 50 pg/mL for the *pagA* gene of *Bacillus anthracis* using PbS. As an alternative to toxic metal-based nanoparticles, Wang et al. reported a strategy for the detection of low levels of *E. coli* using liposome ‘nanocarriers’ loaded with Ca^2+^ ions [55]. Upon the successful formation of a recognition event, calcium-loaded liposomes were bound to the reporter DNA after which they were lysed by a surfactant. In this approach, sub-fmol DNA detection limit was achieved by employing Ca^2+^ ion-sensitive electrodes.

The high surface area of nanoparticles was exploited for loading of a high amount of capture DNA. Chowdhury et al. had detected Dengue virus DNA using nanocomposites of gold nanoparticles (AuNP) with nitrogen and sulfur co-doped graphene quantum dots (N,S-GQDs@AuNP) [56]. First, N,S-GQDs@AuNP were coated with a capture DNA (polydA) using Au-thiol bond formation. This led to the accumulation of a large number of single-stranded (ssDNA) capture DNA. Second, polydA was used to hybridize with a polydT tail of the target viral DNA. Finally, the complex was subjected to electrochemical quantification using methylene blue as a reporter molecule. In the presence of a target, hybridization resulted in a double-stranded DNA (dsDNA), which did not bind methylene blue effectively and resulted in low peak current. In the absence of the target, the dye binds to the capture ssDNA and gave high current in differential pulse voltammetry analysis. This signal-off mode analysis detected a synthetic target in the range of 10 fM to μM. Xu et al. employed AuNPs and exonuclease I for detection of uropathogen’s DNA using [Ru(NH_3_)_6_]^3+^ redox molecules [62]. The AuNPs were coated with multiple ssDNA that was used as the signal probe, this resulted in a greater number of DNA molecules per molecular recognition event. [Ru(NH_3_)_6_]^3+^ bound to the excess DNA molecules electrostatically, which ultimately amplified the redox signal for every target DNA. Furthermore, exonuclease I (Exo I) treatment removed the unhybridized single-stranded capture DNA probes, which minimized the background current. The combination of signal amplification and background current reduction resulted in 1 fM detection limit. Chen et al. reported the use of redox active carbon nanotubes (CNTs) doped with polyaniline (PANI) and endonuclease mediated target recycling approach for detection of *Mycobacterium tuberculosis* [63]. In the presence of target DNA and an assistant probe that hybridized to the capture probe, a hairpin structure has opened to form a Y-shaped junction. Endonuclease recognized the sequence in the Y-shaped junction and released the assistant probe and target DNA. Released target DNA triggered the next cycle of cleavage. After hybridization between CNTs-PANI tagged reporter probe and the cleaved capture probe on the electrode surface, the electrochemical signal of CNTs-PANI was used as a readout. This strategy detected the target in a range between 1 fM to 10 nM.

### 3.3. Nucleic Acid Amplification and Processing Based Approaches

**Figure 4:**
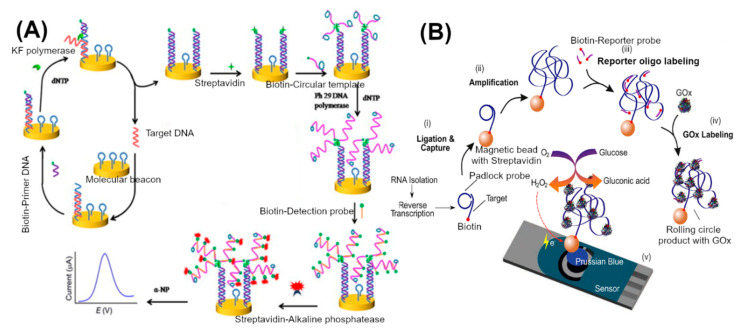
Enhancement of nucleic acid detection by employing polymerase and other isothermal amplification approaches on the electrode surface. (**A**) Strand displacement reaction and rolling circle amplification coupled system [78]. (**B**) Ligation and rolling circle amplification coupled system [68]. Adapted with permission from cited sources.

Though enzyme-based and nanomaterial-based signal amplification approaches reach sensitivity in the femtomolar regime, they still depend on PCR to produce sufficient target DNA. In nucleic acid-based approaches, the enzyme-mediated isothermal amplification of nucleic acids plays an important role in sample amplification and detection. Unlike PCR, which requires specialized thermal cycler instruments to mediate denaturation, annealing and subsequent extension steps, isothermal amplification could be carried out at a constant temperature to produce about a million copies of the target DNA. For isothermal amplification, in addition to DNA polymerase, ligase, nicking enzymes and helicases are employed for specific amplification of target DNA molecules. A detailed review of the method can be found in the following references [76,77]. Simple temperature control makes isothermal amplification an attractive alternative to PCR for point-of-care applications. There are several choices of methods that are available for isothermal amplification, depending on the length, secondary structures and nature of the target (RNA or DNA) [76]. The challenging aspects are electrochemical detection of specific targets amplified by the isothermal method. Cheng et al. reported a method combining circular strand displacement polymerization reaction (CSD), rolling circle amplification (RCA) and enzymatic amplification to enhance the electrochemical sensing of a target DNA (Figure 4A) [78]. First, the capture probe (SH-ssDNA with a hairpin loop structure-molecular beacon) was immobilized on a gold electrode. Second, the strand displacement (CSD) reaction was carried out by adding the target DNA, and biotinylated-primer DNA to the electrode. In the presence of the target DNA, the hairpin structure of the molecular beacon opens and parts the sequence that was hybridized with the target DNA. Another part of the capture probe sequence binds specifically with a biotinylated-primer DNA. The primer sequence was extended towards the target DNA binding region by a DNA polymerase (KF exo-), which led to the release of the target DNA. At this stage, freed target DNA binds to another capture probe to trigger another strand displacement reaction, which results in multiple biotin-tagged DNA duplexes on the electrode surface. This biotin-tagged DNA duplex anchored with another streptavidin-primer specific for rolling circle amplification. Upon addition of specific circular ssDNA templates, deoxynucleotide triphosphates and phi29 DNA polymerase, the RCA reaction produces long ssDNA molecules with tandem repeated sequences. Then alkaline phosphatase tagged reporter probe DNA was added and hybridized with repeated sequences of RCA products. Alkaline phosphatase was used as a final redox signal amplifier at the electrode surface. Using this approach, 1 fM to 100 pM of synthetic target DNA was detected. Huang et al. used a similar strand displacement and rolling circle amplification approach—without biotin and streptavidin tags—for the detection of synthetic DNA sequences specific to the hepatitis B virus [65]. The rolling circle amplification resulted in long ssDNA. They detected the final rolling circle amplification product using methylene blue and reported detection in the range of 10 aM to 0.7 fM. Yanyan et al. reported a DNA detection approach for the avian influenza virus based on isothermal exponential amplification coupled with hybridization chain reaction [67]. Catalytic G-quadruplex–hemin, HRP-mimicking DNAzymes, was tagged to the final molecular recognition complex. Electrochemical signals obtained by measuring the increase in the reduction current of oxidized 3,3′,5,5′-tetramethylbenzidine sulfate, which was generated by DNAzyme in the presence of H_2_O_2_. This method exhibited detection limits of 9.4 fM. In a similar approach, exonuclease III mediated target DNA recycling and G-quadruplex–hemin reported for the detection of HIV gene sequence with a detection limit of 3.6 pM [79].

Ciftci et al. reported a method for tagging multiple glucose oxidase (GoX) enzymes using rolling circle amplification for the detection of *Ebolavirus* (Figure 4B) [68]. In the first step (i) biotinylated primers were used to reverse transcribe the RNA target to cDNA. Then the biotinylated cDNA target hybridized with linear pad-lock probes (PLPs). PLPs have a special sequence feature that renders the probe circular upon hybridization. These circular PLPs are ligated using the enzyme ligase; then biotinylated cDNA target-PLP complex captured on streptavidin-functionalized magnetic beads. Magnetic beads were used for the separation of target DNA from the sample. In the second step, RCA reaction was carried out to produce bulky tandem repeats of DNA coils. In the third step, RCA products were hybridized with the biotinylated-reporter probe, which was then bound to streptavidin-GoX. The final DNA recognition complex was quantified by GoX activity using chronoamperometry. The product, H_2_O_2_, of glucose oxidation by GoX in the presence of oxygen was electrochemically measured. In this method, 1–100 pM of synthetic target DNA was measured. An *Ebolavirus* positive clinical sample was also successfully differentiated from negative samples using this method. A similar Pad-lock-probe approach was demonstrated for the detection of *Ebolavirus* using HRP as a signal amplifier [80].

Helicase dependent amplification (HDA) is another widely employed isothermal system for amplification of target DNA [81]. Helicase is being used to unwind the double-stranded DNA instead of temperature-dependent denaturation during polymerase mediated amplification of the target gene [82]. Barreda-García et al. reported an asymmetric HDA for that resulted in a single-stranded target DNA from *Mycobacterium tuberculosis* [69]. The amplified ssDNA was selectively quantified using enzyme-mediated signal amplification assay. The system was sensitive to 0.5 aM of target DNA. In another study, HDA was also demonstrated for hybridizing the double-stranded target generated from *Salmonella* genome to a single-stranded capture DNA bound to indium-tin-oxide electrodes [83].

Yan et al. reported the detection of pathogenic DNA directly by transcription of RNA from the target DNA [66]. In this approach, hairpin structured primers were designed to open and bind to the target DNA specifically. The primer was extended using DNA polymerase (KF exo-) at 37 °C. The primer has efficiently triggered the circular primer extension reaction, i.e., the resultant dsDNA was further amplified by another primer binding and extension cycles. Additionally, the primer was designed to have a T7 RNA polymerase promoter, which served as a template for in vitro transcription of target DNA. The RNA products from the transcription reaction were directly hybridized with immobilized capture probes. The enzyme tagged signal probe was used to detect the hybridized products. With this approach, *Salmonella*’s *invA* gene from genomic DNA extract was successfully detected. Limit of detection was about 1 fM.

## 4. Conclusions and Future Perspectives

In nanomaterials and enzyme-mediated amplification approaches, DNA/RNA isolation and amplification of targets by PCR are commonly required for real sample electrochemical detection to increase the number of specific target DNA molecules. However, PCR can only be carried out using a specialized thermocycler. Thus, to reduce the dependence on PCR, isothermal amplification methods are explored in clinical sample target amplification in combination with an electrochemical sensor. While isothermal amplification provides the advantage of in situ amplification of target DNA at a constant temperature, more exploration of novel approaches for amplification and hybridization on-electrode surfaces is still needed to achieve practical electrochemical DNA sensors.

The requirement for single-stranded target DNA for hybridization is another constraint for electrochemical DNA sensors. To counter this issue, asymmetric PCR, thermal denaturation of target DNA followed by abrupt cooling, helicase mediated asymmetric DNA amplification, DNA to RNA transcription using RNA polymerase have been employed to yield single-stranded targets. However, novel approaches should be explored for the detection of double-stranded genomic DNA or structured single-stranded cDNA/RNA at room temperature.

Electrochemical analytical methods reached a “glass ceiling” with a limit of detection in the femtomolar concentration range. Enzymes, nanomaterials and molecular tools are successfully engineered for fM detection of DNA on an electrode surface using signal amplification approaches. With this, (i) future progress in the simplification of sample processing steps, (ii) developing approaches to detect double-stranded target DNA and (iii) making advancement in the sensitivity of instrumentation or handheld electrochemical devices will be crucial for achieving practical point-of-care electrochemical devices for pathogen detection in low titers.

In addition, for fast epidemic containment, in order to make sure that highly trained personnel will not be needed for point of care detection, it is highly needed to be able to interface such sensors with already existing simple devices such as glucometers, so it will be easy to sample (such as in non-invasive devices) and simple to read-out the signal for less trained users (e.g., airports personnel).

## Figures and Tables

**Table 1:** Direct oxidation of DNA oxidation of guanine on different electrode supports.

Electrode	Reference Electrode	Electrolyte	Guanine Oxidation Peak (E_p_) (V)	Reference
Gold	Ag/AgCl	PBS, pH 7.4	+0.7/+0.8	[34]
Nafion/Graphene	SCE	0.1 M PBS (pH 4.4)	+0.8	[35]
Glassy carbon electrode	Ag/AgCl	0.1 M PBS (pH 7.0)	+0.6	[36]
Boron doped diamond	Ag/AgCl	0.1 M acetate buffer (pH 4.5)	+0.9	[37]
Pencil graphite	Ag/AgCl	0.5 M acetate buffer and 20 mM LiCIO_4_	+0.76	[38]
DWNTs, and MWNTs	Ag/AgCl	PBS (pH 6)	+1	[39]
HOPGE	Ag/AgCl	0.1 M sodium acetate buffer (pH 7.6)	0.9	[40]

SCE—Saturated calomel electrode, PBS—Phosphate buffered saline, DWNTs—double-walled carbon nanotubes, MWNTs—multi-walled carbon nanotubes, HOPGE—Highly ordered pyrolytic graphite electrode.

**Table 2:** Various signal amplification strategies employed for the detection of pathogenic DNA using electrochemical analytical methods.

Pathogen	Target	Capture Probe	Reporter Probe	Electrode Modification	Amplification Strategy	Redox Signal	Limit of Detection (LOD) *	Analytical Technique	References
*Ebolavirus*	Biotin-ssDNA	HS-ssDNA	NA	Au	Strep-alkaline phosphatase	4-aminophenol	4.7 nM	DPV	[48]
*Avian influenza A* (H7N9) virus	ssDNA	SH-tetrahedral DNA	Biotin-ssDNA	Au	Strep-HRP	TMP	0.75 pM	Amperometric	[49]
Bacteria 16s RNA gene	ssDNA and genomic DNA	ssDNA (polydA SAM)	Biotin-ssDNA	Au	Strep-HRP	TMB	10 fM	Amperometric	[50]
*Zika* virus	ssDNA	Biotin-ssDNA (Strept-magentic beads)	Digoxigenin -ssDNA	Au	Anti- Digoxigenin coupled HRP	TMB	0.7 pM	Chronoamperometry	[51]
HIV DNA	ssDNA	SH-ssDNA	SH-ssDNA	Glucose meter	Invertase-Fe_3_O_4_-Au	Glucose	0.5 pM	Amperometry	[11]
Human *cytomegalovirus*	ssDNA(PCR product)	NA	Biotin-ssDNA	Carbon	Strep-HRP	Ophenyldimine/2,2′-diaminoazobenene	3.6 × 10^5^ copies/mL	DPV	[52]
*E. coli*	gDNA	SH-ssDNA	Biotin-ssDNA	Au	Strep-HRP and redox cycling	*p*-aminophenyl phosphate	0.5416667	Chronoamperometric	[53]
*Lactobacillus brevis*	gDNA, RNA	Biotin-ssDNA	Biotin-ssDNA	Au	Strep-Lipase	Ferrocene	16 amole	CV	[54]
*E. coli*	ssDNA	HS-ssDNA	Biotin-ssDNA	Au	Liposome loaded with Ca^2+^	Ca^2+^ ion-selective electrode (No redox reaction)	0.2 nM	Potentiometric method using	[55]
*Dengue* virus	PCR amplified target with poly (dT)	HS-ssPoly(dA)	Fluro-ssDNA	Au-Polyaniline/N,S-GQDs@AuNP-dA	Nanomaterial as carrier	Methylene blue-intercalation	9.4 fM	DPV	[56]
*Citrus tristeza* virus	ssDNA	HS-ssDNA	NA	Au/AuNPs	Nanoparticle as carrier	[Fe(CN)_6_^3−/4−^]	100 nM	Impedance	[57]
*Chikungunya* virus	ssDNA	ssDNA	NA	Carbon/Fe_3_O_4_@Au (+ and − charge interaction to accumulate the DNA)	Nanoparticle as carrier	Methylene blue	0.1 nM	DPV and CV	[58]
*Human papilloma* virus	ssDNA	HS-ssDNA	NA	Nanoporous polycarbonate-AuNTs	Nanoparticles as carrier	[Fe(CN)_6_^3−/4−^]	1 fM	Impedance	[59]
*Influenza* and *Norovirus*	ssDNA	SH-ssDNA	NA	Pt-Au/Iron Oxide-CNT	Nanoparticles as carrier	NA	8.8 pM	Conductivity (the resistance change)	[60]
*E. coli* uropathogens	ssDNA	Biotin-ssDNA	Biotin-ssDNA	Glassy carbon	CdS quantum dots as reporter	Cd^2+^	0.22 fM	SWV	[61]
*E. coli*	ssDNA	SH-ssDNA	ssDNA	AuNP-deposited on glassy carbon electrode	Nanoparticle as high amount reporter probe carrier	[Ru(NH_3_)_6_]^3+^	1 fM	DPV	[62]
*Mycobacterium tuberculosis*	PCR product	SH-ssDNA	SH-ssDNA loaded AuNPs@ CNT-PANI	Au	Endonuclease	Polyaniline	0.33 fM	DPV	[63]
*Enterobacteriaceae*	ssDNA and HAV cDNA	HS-ssDNA	biotin-ssDNA	Au	Exonuclease III and Strep-alkaline phosphatase	*α*-naphthyl phosphate	8.7 fM	DPV	[64]
*Hepatitis B* virus (HBV)	ssDNA	HS-ssDNA	ssDNA as primer	Au	CSD and RCA	Methylene blue	2.6 aM	DPV	[65]
*Salmonella*	gDNA	SH-ssDNA	Biotin-ssDNA	Au	DNA polymerase, T4 RNA polymerase and Strep-alkaline phosphatase	α-naphthyl phosphate	0.97 fM	DPV	[66]
*Avian influenza A* (H7N9) virus	ssDNA	SH-ssDNA	Molecular beacons	Au	EXPAR-HCR and G-quadruplex–hemin-(HRP like catalysis)	TMB	9.4 fM	DPV	[67]
*Ebolavirus*	RNA	cDNA synthesized from target RNA	Biotin-ssDNA	Carbon	RCA and Strep-glucose oxidase	H_2_O_2_	1 pM	Chronoamperometry	[68]
*Mycobacterium tuberculosis*	gDNA	Biotin-PCR product from target	Fluorescein-ssDNA	Carbon	HDA and Antifluorescein-POD Fab	TMP	0.5 aM	Chronoamperometry	[69]

DPV—Differential pulse voltammetry, CV—Cyclic Voltammetry, EIS—Electrochemical Impedance spectroscopy, CSD—Circular strand displacement, RCA—Rolling circle amplification, EXPAR—Isothermal exponential amplification, HCR—Hybridization chain reaction, HDA—Helicase dependent amplification, TMB—3,3′,5,5′-tetramethylbenzidine, N,S-GQDs@AuNP—Nitrogen, sulfur codoped graphene quantum, CNT-PANI—Carbon nanotube-polyanilline, NA—Not applicable, * If limit of detection is not reported, lowest detected value is provided. ssDNA—Single stranded DNA.
